# *NRN1* Gene as a Potential Marker of Early-Onset Schizophrenia: Evidence from Genetic and Neuroimaging Approaches

**DOI:** 10.3390/ijms23137456

**Published:** 2022-07-05

**Authors:** Carmen Almodóvar-Payá, Maria Guardiola-Ripoll, Maria Giralt-López, Carme Gallego, Pilar Salgado-Pineda, Salvador Miret, Raymond Salvador, María J. Muñoz, Luisa Lázaro, Amalia Guerrero-Pedraza, Mara Parellada, María I. Carrión, Manuel J. Cuesta, Teresa Maristany, Salvador Sarró, Lourdes Fañanás, Luis F. Callado, Bárbara Arias, Edith Pomarol-Clotet, Mar Fatjó-Vilas

**Affiliations:** 1 FIDMAG Germanes Hospitalàries Research Foundation, 08830 Sant Boi de Llobregat, Barcelona, Spain; calmovodar@fidmag.com (C.A.-P.); mguardiola@fidmag.com (M.G.-R.); psalgado@fidmag.com (P.S.-P.); rsalvador@fidmag.com (R.S.); aguerrero.hbmenni@hospitalarias.es (A.G.-P.); ssarro@fidmag.com (S.S.); 2 Instituto de Salud Carlos III, Biomedical Research Network in Mental Health (CIBERSAM), 28029 Madrid, Madrid, Spain; smiret@gss.cat (S.M.); llazaro@clinic.cat (L.L.); parellada@hggm.es (M.P.); lfananas@ub.edu (L.F.); lf.callado@ehu.eus (L.F.C.); barbara.arias@ub.edu (B.A.); 3 Departament de Psiquiatria, Hospital Universitari Germans Trias i Pujol (HUGTP), 08916 Badalona, Barcelona, Spain; mariagiralt.germanstrias@gencat.cat; 4 Departament de Psiquiatria i Medicina Legal, Universitat Autònoma de Barcelona (UAB), 08193 Bellaterra, Barcelona, Spain; 5 Department of Cell Biology, Molecular Biology Institute of Barcelona (IBMB-CSIC), 08028 Barcelona, Barcelona, Spain; cggbmc@ibmb.csic.es; 6 Centre de Salut Mental d’Adults de Lleida, Servei de Psiquiatria, Salut Mental i Addiccions, Hospital Universitari Santa Maria de Lleida, 25198 Lleida, Lleida, Spain; 7 Institut de Recerca Biomèdica (IRB), 25198 Lleida, Lleida, Spain; 8 Complex Assistencial en Salut Mental Benito Menni, 08830 Sant Boi de Llobregat, Barcelona, Spain; mmunoz.hbmenni@hospitalarias.es; 9 Department of Child and Adolescent Psychiatry and Psychology, Institute of Neurosciences, Hospital Clinic de Barcelona, 08036 Barcelona, Barcelona, Spain; 10 Departament de Medicina, Universitat de Barcelona (UB), 08036 Barcelona, Barcelona, Spain; 11 Institut d’Investigacions Biomèdiques August Pi i Sunyer (IDIBAPS), 08036 Barcelona, Barcelona, Spain; 12 Servicio de Psiquiatría del Niño y del Adolescente, Hospital General Universitario Gregorio Marañón, 28007 Madrid, Madrid, Spain; 13 Instituto de Investigación Sanitaria del Hospital Gregorio Marañón (IiSGM), 28007 Madrid, Madrid, Spain; 14 Departamento de Psiquiatría, Facultad de Medicina, Universidad Complutense, 28040 Madrid, Madrid, Spain; 15 Hospital Sant Rafael, 08035 Barcelona, Barcelona, Spain; micarrion.hsrafael@hospitalarias.es; 16 Servicio de Psiquiatría, Hospital Universitario de Navarra, 31008 Pamplona, Navarra, Spain; mj.cuesta.zorita@navarra.es; 17 Instituto de Investigación Sanitaria de Navarra (IdiSNA), 31008 Pamplona, Navarra, Spain; 18 Departament de Diagnòstic per la Imatge, Hospital Sant Joan de Déu Fundació de Recerca, 08950 Esplugues de Llobregat, Barcelona, Spain; tmaristany@sjdhospitalbarcelona.org; 19 Departament de Biologia Evolutiva, Ecología i Ciències Ambientals, Universitat de Barcelona (UB), 08028 Barcelona, Barcelona, Spain; 20 Institut de Biomedicina de la Universitat de Barcelona (IBUB), 08028 Barcelona, Barcelona, Spain; 21 Department of Pharmacology, University of the Basque Country, UPV/EHU, 48940 Leioa, Bizkaia, Spain; 22 Biocruces Bizkaia Health Research Institute, 48903 Barakaldo, Bizkaia, Spain

**Keywords:** schizophrenia-spectrum disorders, *NRN1*, age at onset, working memory, functional magnetic resonance imaging (fMRI)

## Abstract

Included in the neurotrophins family, the Neuritin 1 gene (*NRN1*) has emerged as an attractive candidate gene for schizophrenia (SZ) since it has been associated with the risk for the disorder and general cognitive performance. In this work, we aimed to further investigate the association of *NRN1* with SZ by exploring its role on age at onset and its brain activity correlates. First, we developed two genetic association analyses using a family-based sample (80 early-onset (EO) trios (offspring onset ≤ 18 years) and 71 adult-onset (AO) trios) and an independent case–control sample (120 healthy subjects (HS), 87 EO and 138 AO patients). Second, we explored the effect of *NRN1* on brain activity during a working memory task (N-back task; 39 HS, 39 EO and 39 AO; matched by age, sex and estimated IQ). Different haplotypes encompassing the same three Single Nucleotide Polymorphisms(SNPs, rs3763180–rs10484320–rs4960155) were associated with EO in the two samples (GCT, TCC and GTT). Besides, the GTT haplotype was associated with worse N-back task performance in EO and was linked to an inefficient dorsolateral prefrontal cortex activity in subjects with EO compared to HS. Our results show convergent evidence on the *NRN1* association with EO both from genetic and neuroimaging approaches, highlighting the role of neurotrophins in the pathophysiology of SZ.

## 1. Introduction

Substantial evidence highlights the importance of the genetic component in the aetiology of schizophrenia (SZ), with an estimated heritability of around 65–79% [1,2]. Indeed, genome-wide association studies (GWAS) have confirmed SZ’s polygenic architecture, resulting from the aggregated effect of low impact variants and reporting an SNP-based heritability of 24% [3]. Moreover, genomic data converge into identifiable biological pathways involved in neurodevelopment, particularly highlighting the mechanism of synaptic plasticity [3,4,5]. 

However, in the search for specific genetic factors related to SZ, studies face several challenges that arise from the genetic and phenotypic complexity of the disorder [6]. Then, it has been suggested that combining complementary designs, such as family-based and case–control, would clear the way to dissect the genetic influences of the disorder [7]. In this sense, family-based genetic association designs have the advantage of reducing the problem of stratification and spurious association when compared to case–control studies, while the latter usually allow for larger sample sizes [8]. 

In addition to the design, the approaches to the phenotypic complexity of SZ have also been considered using narrower phenotypes with particular aetiological significance to reduce the heterogeneity and identify specific genetic factors associated with the disorder. One of these phenotypes is the age at onset, which shows a heritability of around 33% [9]. Notably, early age at onset (EO) has captured much attention because it is considered a marker of a higher genetic liability than adult-onset (AO) [10,11]. The EO term includes cases with onset up to 18 years of age and, despite being arbitrary, roughly corresponds with the upper age cut-off in most published studies of child and adolescent psychosis [12]. In this support, EO subjects show a higher familial aggregation of SZ and other mental disorders [13], poorer premorbid adjustment [14] and neurocognitive performance [15], more severe outcomes [16,17] and more prominent alterations in neurodevelopmental trajectories than AO forms [18]. The few GWAS focused on searching for genetic loci associated with age at onset in SZ have confirmed that some variants overlap with those conferring risk for SZ, while others are pure modifiers [19,20,21,22]. Remarkably, EO patients present higher SZ polygenic risk scores than their siblings, with the scores effectively predicting an earlier age at onset [23]. Interestingly, the variants associated with an earlier age at onset converge into molecular networks related to nervous system development, the regulation of axon extension, modulation of glial proliferation, molecular transport, and cell-to-cell signalling and interactions [20,22]. 

Among genes with pivotal roles through all stages of the brain’s formation, there is the Neuritin 1 gene (*NRN1*, 6p25.1) (see review [24]), which is highly expressed in the hippocampus, the cerebral cortex and the cerebellum [25,26] in an activity-dependent manner [27,28]. Although the Nrn1 receptor and its downstream signalling effectors are still being studied, it seems that Nrn1 regulates synaptic excitability through the activation of the insulin receptor (IR) and its downstream signalling pathways [29,30]. Consequently, inadequate Nrn1 sustenance could translate into the abnormal formation of synapses, a reduced capacity to perform adaptive responses and, in turn, a higher risk of developing a mental disorder. In fact, the interest in the role of Nrn1 in SZ has been motivated by several studies, which have evidenced its impact on cognitive function through synaptic plasticity mechanisms. From cell- and animal-based approaches, it has been shown that the viral-mediated overexpression of *NRN1* in different models (unpredictable stress-induced rat depression model, mice exposed to low-frequency electromagnetic fields and an Alzheimer’s disease model Tg2576 mouse) prevents the atrophy of dendrites and spines and improves associated behaviours, such as anxiety, depression, deficits in novel object recognition, learning and memory [31,32,33]. Additionally, the expression of *NRN1* has been shown to increase in the hippocampus of mice exposed to electroconvulsive therapy and fluoxetine administration [32,34]. These studies highlight the potential therapeutic use of *NRN1* in disorders associated with loss of cognitive function, such as SZ, and appeal for a better understanding of its molecular mechanisms. From human-based studies, *NRN1* has been already defined as a candidate gene for SZ since specific allelic variants have been associated with an incremented risk of developing the disorder. Moreover, *NRN1* has also been described as a modifier of the SZ phenotype due to its association with patients’ general cognitive ability and age at onset [35,36]. This suggests that *NRN1* may be involved in critical mechanisms of brain development, particularly in those most susceptible to the earlier onset of the symptoms.

Neuroimaging data can provide evidence on how the genetic actors underlying an earlier age at onset contribute to the neurobiology of the disorder [37]. In this sense, functional neuroimaging studies focused on exploring the brain activity during working memory (WM) tasks (related to the capacity to retain and use mental items during a short period) are of particular interest. Subtle WM deviances have been described in the healthy siblings of subjects with SZ compared to healthy subjects (HS) in studies focused on cognition [38], brain activity [39] and connectivity [40]. This suggests that WM alterations in SZ are genetically influenced. Indeed, disabilities in this cognitive domain are considered to be a core feature of SZ [41] and have been reported to be even more severe in EO patients [15]. 

Several studies based on functional magnetic resonance neuroimaging (fMRI) and exploring brain networks supporting WM have consistently described frontoparietal differences in individuals with SZ when compared to HS. Most of these studies described the decreased activity of the dorsolateral prefrontal cortex (DLPFC), the ventrolateral prefrontal cortex (VLPFC) and anterior cingulate cortex (ACC) as a key mechanism of WM dysfunction [42]. The few functional neuroimaging studies specifically focused on individuals with EO have reported similar patterns of abnormal activations in these regions of the prefrontal cortex (e.g., VLPFC, DLPFC, and ACC) plus some limbic and temporal regions [43,44,45,46,47,48]. However, those studies are scarce, in part, due to the low rate of EO, which represents only about 8% among individuals with SZ [49], and they have reported inconsistent findings regarding the direction of the results. In this context, the study of the genetic WM correlates in individuals with EO forms is particularly pertinent since it could offer insights into the impact of genetic architecture on brain activity and, ultimately, on the clinical manifestation of SZ. 

Considering all the above-cited evidence, we hypothesised that the polymorphic variability of *NRN1* would be differentially associated with the risk of developing EO forms of SZ compared to AO. We developed this study by combining different designs (family-based and case–control sample approaches) to provide robustness to our findings. Additionally, we hypothesised that those genetic variants conferring risk for EO would differentially impact WM-related brain activity.

## 2. Results

### 2.1. Genetic Association Analyses 

#### 2.1.1. Family-Based 

The genotypes/alleles counts and frequencies of EO/AO offspring and parents are listed in Supplementary Table S1. As shown in Table 1, within EO families, the GCT haplotype including SNP6, SNP7 and SNP8 (HAP678) was significantly under-transmitted from parents to affected offspring (*p_perm_* = 0.03). Our analyses did not reveal any association between the genetic variability at *NRN1* with the risk for AO SSD, neither in the allelic, genotypic or haplotype approach.

#### 2.1.2. Case–Control 

The distribution of the genotypes/alleles in HS, EO and AO subjects is reported in Supplementary Table S2. As exposed in Table 1, we observed a significant association of SNP6 G allele (*p_perm_* = 0.02), SNP7 T allele (*p_perm_* = 0.03) and SNP8 T allele (*p_perm_* = 0.02) with EO SSD under an additive model. We also identified an association of two haplotypes including SNP6, SNP7 and SNP8 (HAP678) with the risk for EO SSD, which was in line with the SNP-based results. The GTT haplotype was significantly more frequent in subjects with EO SSD than in HS (*p_perm_* = 0.02), while the TCC was more frequent in HS (*p_perm_* = 0.01). Other 2-SNP and 4-SNP haplotypes containing these same variants were also associated with the risk for EO (Supplementary Table S3). Our analyses did not reveal any effect of genetic variability at *NRN1* on the risk for AO SSD, in any of the tested models (allelic, genotypic and haplotypic).

### 2.2. Neuroimaging Genetic Association Analyses

#### 2.2.1. N-Back Functional Response

The three groups (HS, EO and AO) showed typical WM-related activation and deactivation patterns (Supplementary Figures S1–S3). In addition, both EO and AO exhibited a deactivation failure when compared to HS in overlapping regions involving bilateral structures, such as the frontal gyrus (superior, medial and inferior orbital part), the olfactory area, the rectus and the anterior cingulate and the paracingulate gyri, as well as right structures, such as the superior and middle temporal gyrus, the parahippocampal gyrus, the hippocampus, the amygdala, the fusiform gyrus and the caudate nucleus (Supplementary Figure S4).

**Figure 1:**
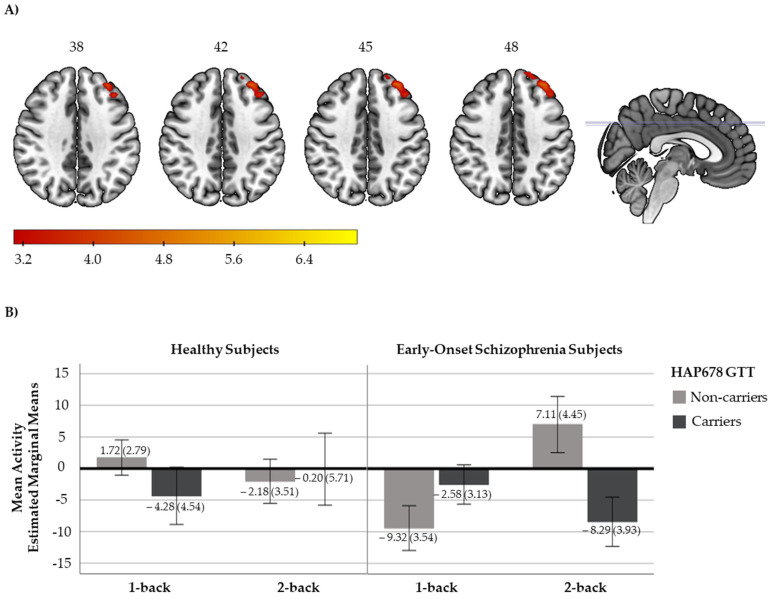
(**A**) Axial view of the brain showing the significant cluster derived from the diagnosis x *NRN1* HAP678 GTT analysis in the 2-back vs. 1-back contrast. A sagittal view with the marks of the cross slices is also included. The right side of the image represents the right side of the brain. The MNI coordinates are given for the shown slices. Units of the bar correspond to the β values of the regression, standardised to Z scores. (**B**) Bar plots with the cluster mean activity (estimated marginal means and ±2 standard errors (se)) for healthy subjects (HS; left, non-carriers: n = 27, carriers: n = 10) and subjects with early-onset schizophrenia (EO; right, non-carriers: n = 19, carriers: n = 20).

We detected a significant diagnosis x HAP678 (GTT) interaction for the EO vs. HS comparison in the 2-back vs. 1-back contrast in one cluster located at the superior and middle frontal gyrus, regions of the DLPFC (316 voxels, peak activation at MNI coordinates [−34,42,42], Zmax = 4.54, *p* = 0.0025, Figure 1A). To further interpret this result, mean activity scores for the 1-back and 2-back contrasts were plotted. As shown in Figure 1B, HS exhibited a cluster mean activity of around zero for the two contrasts, irrespective of their haplotypic profile. Subjects with EO without the risk haplotype showed a pattern towards increased cluster activity from 1-back to 2-back contrasts, whereas those patients carrying the risk haplotype presented a pattern towards decreased cluster activity. We did not observe any significant interaction on brain activity when we compared HS and AO groups. 

#### 2.2.2. N-Back Behavioural Response

First, subjects with EO exhibited a globally poorer performance of the N-back task than HS in both difficulty levels (mean (SD) d’1: EO 3.07 (1.16) and HS 4.14 (0.68), F = 13.00, *p* = 0.001; mean (SD) d’2: EO 2.06 (0.90) and HS 3.41 (0.88), F = 27.52, *p* < 0.001). While both groups showed different scores at the two levels of the task, their degree of decrease in performance from the 1-back to 2-back was similar (F = 1.55, *p* = 0.22).

Second, AO and HS exhibited a similar performance in the low memory load condition, but their performance diverged in the high memory load condition (mean (SD) d’1: AO 3.81 (0.90) HS 4.14 (0.68), F = 0.33, *p* = 0.57; mean (SD) d’2: AO 2.48 (0.82) and HS 3.41 (0.88), F = 15.27, *p* < 0.001). Then, as the performance of the two groups was similar for the 1-back, the degree of change from the 1-back to -back was more pronounced in subjects with AO than HS (F = 8.11, *p* = 0.01). 

**Figure 2:**
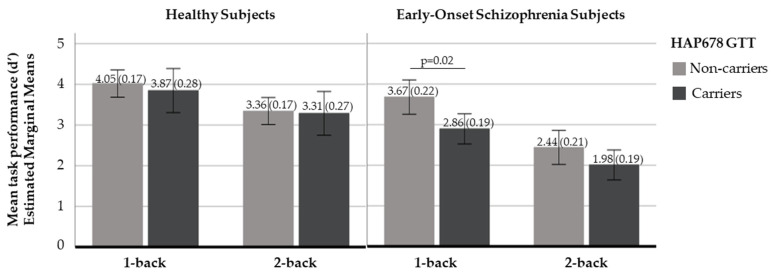
Bar plots with mean performance (estimated marginal means and ±2 standard errors(se)) for healthy subjects (HS; left, non-carriers: n = 27, carriers: n = 10) and subjects with early-onset schizophrenia (EO; right, non-carriers: n = 19, carriers: n = 20) by *NRN1* HAP678 GTT.

Third, EO performance at the low memory load condition was modulated by *NRN1* haplotypic variability. Subjects carrying the HAP678 GTT showed a poorer performance when compared to those without the risk haplotype (mean (SD) d’1: non-carriers 3.67 (0.22) and carriers 2.86 (0.19), F = 5.66, *p* = 0.02) (Figure 2). No effect of *NRN1* haplotypic variability on task performance was detected in either HS or AO subjects.

## 3. Discussion

In this study, we combined genetic association and neuroimaging approaches to deepen into the role of *NRN1* in the age at onset of SZ. Regarding the genetic association approach, our study adds to the only two previous studies on the association of the *NRN1* gene with SZ and other disorders within the spectrum [35,36], but it is the first to be developed through family-based and case–control designs in two independent samples. Our results, derived from the two samples, suggest that the variability at *NRN1* may explain a modest proportion of the risk of EO. Concerning the neuroimaging approach, our study represents the first to explore WM neural correlates of *NRN1*. Our findings indicate that *NRN1* variants conferring risk for SZ also have an effect on the performance of the N-back task, specifically within EO subjects. Additionally, we report brain activity differences between EO subjects and HS located at the DLPFC conditional to the same genetic variants.

Our genetic association analyses identified different SNPs and haplotypes at *NRN1* associated with EO in SSD. We detected a significant under-transmission of the HAP678 GCT from parents to affected offspring, specifically in EO families. In parallel, through a case–control approach, we identified the effect of SNP6, 7 and 8 on the risk for EO SSD and the association of a risk haplotype encompassing these same polymorphisms HAP678 GTT. On the contrary, we did not detect any association with AO SSD. Therefore, our data converge into the view of polymorphisms at *NRN1* (SNP6, SNP7, SNP8) as a relevant genetic variability source in modifying the neurodevelopment processes related to the earlier emergence of these disorders. It is of note that these SNPs have been previously associated with the risk for SZ in the two-preceding works [35,36]. Moreover, one of these studies also identified a role of *NRN1* in age at onset of SSD [36]. However, our results should be interpreted in the context of the polygenic architecture of these disorders, as the effect of the SNPs and haplotypes is small (see the corresponding ORs). Still, this evidence suggests that those genes that influence brain development, such as *NRN1*, may modify illness traits, such as age at onset, and ultimately affect the risk for these disorders.

Due to the few studies focused on examining the association of the *NRN1* gene with SZ, data from whole-genome approaches must be taken into consideration for the further interpretation of our results. First, different genetic linkage studies mapping SZ to a genomic location pointed towards the association of chromosome region 6p24-25 and highlighted *NRN1* as a positional candidate gene [50,51,52,53]. However, as far as we know, *NRN1* has not appeared as a significant locus in the latest genome-wide association studies [3]. These negative results could be explained due to the modifier properties of *NRN1*, which means that, as our results suggest, *NRN1* modulates SSD phenotype through its impact on age at onset. Some linkage and whole-genome studies that specifically aimed to identify modifier loci related to the age at onset in SZ have highlighted the chromosome region 6p24 and some *NRN1* neighbouring intergenic variants with putative regulatory roles on its expression [21,54]. Additionally, whole-genome approaches have also linked *NRN1* and SZ through epigenetic mechanisms. In this respect, Pidsley et al., 2014 [55] identified, through a methylomic approach in human post-mortem prefrontal cortex samples, a wide genetic region that is hypomethylated in patients compared to controls, spanning the body of *NRN1*. This result suggests that *NRN1* could be differentially expressed in the prefrontal cortex of subjects with SZ, a brain region repetitively described to be altered in this disorder [56]. 

To explore the neurobiological translation of the observed genetic variants conferring a higher risk for EO SZ, we developed a neuroimaging genetic study in a matched case–control sub-set. Our functional data suggest that the risk haplotype (HAP678 GTT) that is associated with the earlier emergence of the disorder is also associated with DLPFC activity changes within this group of patients. Concretely, through the analysis of differences between the two levels of the N-back task (2-back vs. 1-back contrast), we observed that EO subjects not carrying the risk haplotype changed DLPFC activity towards activation in response to the task’s increasing difficulty. At the same time, those carrying the risk haplotype were prone to decreased activity. The previous few studies exploring whole-brain activity differences between subjects with EO and HS have reported inconsistent findings regarding the implicated regions. Some reported reduced activation of the left VLPFC and extrastriate visual cortex [43], while others described VLPFC hyperactivation [47]. Other works reported the reduced engagement of the DLPFC, the ACC, frontal operculum and inferior and posterior parietal and caudate [44,45,46], contrary to other investigations that suggested increased activations in the ACC, medial temporal lobe structures, the insula and bilateral lateral temporal lobes [48]. In this respect, our results shed light on those controversial findings, as they provide evidence that genetic factors, in this case, the *NRN1* gene, could be underlying these differences in DLPFC activity. 

To interpret our functional results in the DLPFC, it is important to integrate brain activity findings with N-back behavioural data. On the one hand, the sustained activation of the prefrontal circuits is considered a key mechanism for executing high-memory-load tasks [57]. On the other hand, it is also known that the degree of change in DLPFC activity is related to the cognitive effort needed to perform the task. In other words, if the computational cost is unlikely to result in the accurate performance of the task, the prefrontal resources get disengaged [58]. In this sense, EO subjects not carrying the risk haplotype displayed a better performance at low memory load and exhibited a higher degree of DLPFC modulation towards activation in response to a higher memory load level. This result suggests that EO subjects without the risk haplotype may use greater prefrontal resources in response to task difficulty increase than those with the risk haplotype, who seem to reach activation and performance peaks at a lower processing load. 

On the whole, our functional and behavioural results align with the preceding evidence linking *NRN1* and cognitive performance in SZ [35,36] and executive function in HS [59]. Furthermore, they suggest that these prefrontal networks of sustained activation during WM, in which *NRN1* appears to have a relevant role, might be especially sensitive to the earlier onset of psychosis. The specific effects in EO forms of SZ seem reasonable since several investigations have described adolescence as a crucial period for the development of the prefrontal cortex [60] and the reorganisation of the WM network [61,62]. This is also supported by several studies showing the greater recruitment of WM regions in adults than in children [63]. In this view, the earlier onset of the disorder might strongly impact the neural trajectories associated with WM development, potentially leading to WM-characteristic impairments in EO compared to AO [64]. Interestingly, regarding the specific role of DLPFC activity and *NRN1* as potential markers of EO, a recent study using an innovative transcriptomic approach defined two molecularly distinct subgroups of subjects with SZ [65]. The first presented a DLPFC transcriptome very similar to that of HS, while the second exhibited a strikingly different DLPFC transcriptome, with the *NRN1* gene included among the differentially expressed genes. These data suggest that fundamental biologic differences exist between subjects diagnosed with SZ. Thus, our results on the modulation effect of *NRN1* haplotypic variability on brain function and performance contribute to bridging the gap between the role of *NRN1* in synaptic plasticity processes and the pathophysiological mechanisms underlying SZ.

Towards a further understanding of such mechanisms, considering the putative effects of the analysed polymorphic sites on gene expression regulatory mechanisms represents a valuable resource to provide additional meaning and importance to our association data. Among the three variants encompassed by the HAP678 risk haplotype (LD = 0.99 in both samples), data from the RegulomeDB and Haploreg highlight the functional effects of SNP6 (rs3763180). There is evidence that this variant could modify the histone enhancer and promoter marks in the brain, contributing to the chromatin state at this locus. Moreover, this variant is predicted to alter motifs that overlap the recognition sequence of different transcription factors, such as the alpha isoform of the CCAAT-enhancer binding proteins (C/EBP), PBX homeobox 3 (PBX3) and the Neuron-Restrictive Silencing Factor (NRSF). Interestingly, the change of a T to a G in that position is linked to increased NRSF affinity, implicated in the programming of stress-sensitive neurons by neonatal experience through epigenetic mechanisms, promoting resilience to stress-related emotional disorders [66]. The other two variants included in the HAP678 presented a lower functionality score; still, data show their putative modulatory effects on the affinity of some transcription factors. For instance, the SNP7 (rs10484320) is suggested to modify the binding of the TATA-binding protein (TBP) and PU.1. The TBP has been associated with the risk for SZ, age at onset and prefrontal function [67]. Additionally, higher levels of the transcription factor PU.1, required for the development of the immune system, have been detected in post-mortem brain samples from individuals diagnosed with SZ compared to HS [68]. Additionally, accordingly to Brainiac data, when the effect of these three SNPs stratifies the expression of *NRN1* transcripts, genotype-based differences emerge in the hippocampus, and a trend effect is detected in the cortex. These lines of evidence suggest putative molecular mechanisms by which the SNPs included in the HAP678 may affect the complex phenotype of SZ. Nevertheless, further functional data on these SNPs are needed to fully characterise their impact on the underlying mechanisms that connect *NRN1* and age at onset of psychosis.

Finally, our study should be interpreted in the context of some limitations. First, regarding our genetic association approach, the samples could be considered to be relatively small. However, according to the statistical power of our analyses and after multiple testing correction procedures, we concurrently identified, in two independent samples, the impact of *NRN1* genetic variants on the risk for the earlier onset of SZ. Second, all the variants included in the present study are polymorphic; however, it is known that a certain proportion of the variance in genetic liability of SZ is also accounted for by rare variants [69,70]. Therefore, different approaches analysing the combined role of common and low-frequency variants along *NRN1* gene on SZ would be of potential interest. Third, while the present study has not directly analysed the functional consequences of the *NRN1* variants associated with EO, our results and the available functional data suggest the need for cell-based studies integrating genetic variability information. Fourth, in the case of neuroimaging approaches, although we compared EO subjects and HS, patients were scanned in their adulthood, years after the onset of the illness. Therefore, illness duration and related clinical variables could have affected the results. Based on this, we checked the possible impact of illness duration or medication on the mean activity and the d’ scores through regressions. While we cannot completely rule out the effect of these variables the lack of significance suggests that our results are not modulated by them. Additionally, it should be underlined that activation differences at prefrontal regions have been observed in unaffected first relatives of SZ patients [39], individuals at clinical high risk for psychosis [71] and individuals with treatment-naïve first episode psychosis [72], suggesting that this pattern may represent an intrinsic feature of SZ rather than a medication effect. Lastly, the absence of representation of diverse ethnic groups and the low proportion of females within our EO group hampers the extrapolation of our results and demands the need for new studies in larger samples with equal representation of those populations. 

## 4. Materials and Methods

### 4.1. Sample

This study included 798 individuals (Table 2). Two independent samples were used to develop separate genetic association analyses: (i) Sample 1 comprised 151 trios (with an offspring diagnosed with schizophrenia spectrum disorders (SSD) plus 302 healthy parents), (ii) Sample 2 consisted of 225 independent patients diagnosed with SSD and 120 HS. Also, from Sample 2, a sub-set of cases with SZ (39 EO and 39 AO) and HS (39) (matched by sex, age and estimated IQ) was selected to develop a neuroimaging genetic analysis (Sample 3). Participants were drawn from admissions to both Child and Adolescent and Adult Psychiatric Units. All HS were recruited from non-medical staff working in the hospital, their relatives and acquaintances, plus independent sources in the community.

All patients were evaluated by experienced psychiatrists and met the DSM-IV-TR criteria for SSD, including schizophrenia, schizophreniform disorder, schizoaffective disorder and psychosis disorder not otherwise specified (Table 2). Patients up to 17 years old were diagnosed following Kiddie Schedule for Affective Disorders and Schizophrenia (KSDAS, [73]), while the Comprehensive Assessment of Symptoms and History (CASH, [74]) or the Structured Clinical Interview for DSM Disorders (SCID, [75]) was used for adult patients. Age at onset of the first episode was determined using these clinical schedules and/or the Symptom Onset in Schizophrenia inventory (SOS, [76]). Following previous studies [12], subjects with SSD were classified as either EO when the first episode occurred before or at 18 years, or as adult-onset AO when presented at age 19 or older. 

The general exclusion criteria included an age above 65 years, major medical illnesses that could affect brain functions, substance-induced psychotic disorder, neurological conditions and having had at least one parent not from European ancestry. Moreover, all the relatives and HS underwent a clinical interview on personal and/or familial psychiatric history using Family Interview for Genetic Studies (FIGS) [77] and those who reported a personal history of mental illness or treatment with psychotropic medication were excluded.

For the subjects included in the neuroimaging study, the exclusion criteria also included an estimated IQ under 70, left manual dominance, and a history of head trauma with loss of consciousness. The evaluation of patients comprised the Positive and Negative Symptoms Scale (PANSS), while the estimated IQ of both patients and controls, was assessed using the Word Accentuation Test [78], which requires the pronunciation of 30 low-frequency Spanish words whose accents were removed. 

All participants provided written consent after being informed about the study procedures and implications. In the case of patients below the age of 18, written consent was also obtained from their parents. The study was performed following the guidelines of the institutions involved and was approved by the local ethics committee of the centre. All procedures were carried out according to the Declaration of Helsinki.

### 4.2. Genotyping 

Genomic DNA was obtained for all individuals either from buccal mucosa through cotton swabs or from peripheral blood cells by punction and extracted using an ATP Genomic DNA Mini Kit Tissue (Teknokroma Analítica, S.A., Sant Cugat del Vallès, Barcelona, Spain) or using a Realpure SSS Kit for DNA Extraction (Durviz, S.L.U, Valencia, Spain), respectively. 

All Single Nucleotide Polymorphisms (SNPs) were determined via a fluorescence-based allelic discrimination procedure (Applied Biosystems Taqman 5′-exonuclease assays) using standard conditions. 

The information about the SNPs is given in Table 3. The SNPs were selected based on two previous studies [35,36]. All SNPs had a minor allele frequency above 5% and were non-coding. As previous evidence suggests that non-coding variants exert important regulatory effects [79] and that such effects are particularly important in SZ [80], the functional consequences of the analysed SNPs were evaluated using different resources. First, HaploReg was used to obtain information about the impact of non-coding variants on chromatin state, protein binding, sequence conservation across mammals, regulatory motifs and expression (https://pubs.broadinstitute.org/mammals/haploreg/haploreg.php, [81], accessed on 1 June 2022). It showed that several SNPs (from rs12333117 to rs3763180) are classified as genetic promoters or enhancers in the brain tissue, based on histone marker data from the Epigenetic Roadmap. Moreover, all the variants, except for one (rs582186), are predicted to change the affinity of multiple regulatory motifs based on data from the ENCODE project. Second, the Regulome DataBase (http://www.regulomedb.org/, [82], accessed on 1 June 2022), a model integrating functional genomic features, was used to obtain a functional probability score for each SNP. This score ranges from 0 to 1, with 1 being most likely to be a regulatory variant [83]. As shown in Table 3, several of the selected SNPs had a score above 0.61 (from rs12333117 to rs3763180). Third, the Brain eQTL Almanac (Braineac), which is a web-based (http://www.braineac.org, accessed on 1 June 2022) resource to access the UK Brain Expression Consortium (UKBEC) dataset, showed that the assessed *NRN1* transcripts are mainly expressed in the cortex, hippocampus and cerebellum. In addition, this tool was also used to evaluate the effect of the SNPs associated with EO forms of SZ on brain expression patterns (see Discussion).

All markers were in Hardy–Weinberg equilibrium in the two samples: the family-based (parents and offspring) and the case–control (subjects with SSD and HS). The total genotypic call rate was 95.48 and 98.85%, respectively.

### 4.3. fMRI Task Description and Acquisition Parameters 

#### 4.3.1. N-Back Task

Functional images were acquired while participants performed a sequential-letter version of the N-back task, which engages storage and executive processes related to attention and WM. Briefly, in this task, letters were presented sequentially in a random way, and the participants were required to press a button when the letter shown on the screen matched the one presented one step prior in the sequence (condition 1-back) or the one from two steps before in the sequence (condition 2-back). The two levels of memory load were presented in a block design manner. Each block consisted of 24 letters shown every 2 s (1 s on, 1 s off), and all blocks contained 5 letter repetitions located randomly within the blocks. Four 1-back and four 2-back blocks were presented in an interleaved way, and between them, a baseline stimulus (an asterisk flashing with the same frequency as the letters) was presented for 16 s. Letters were displayed in green for 1-back blocks and in red for 2-back blocks to identify which condition had to be performed. All participants went through a training session before entering the scanner.

#### 4.3.2. N-Back Performance Data

To measure the behavioural performance of the task, we used the signal detection theory index sensitivity, d’ scores [84]. Higher values of d’ indicate a better ability to discriminate between targets and distractors, while negative values indicate that subjects are not performing the task. Then, all the individuals included in the analyses had positive d’ values (d’1 for 1-back and d’2 for 2-back). 

#### 4.3.3. fMRI Acquisition Parameters

The fMRI data acquisition was performed with a GE Sigma 1.5-T scanner (General Electric Medical Systems, Milwaukee, WI, USA) at Hospital Sant Joan de Déu (Barcelona, Spain). Functional images included 266 volumes for each individual and a gradient echo-planar imaging sequence depicting the blood oxygen level-dependent (BOLD) signal. Each volume contained 16 axial planes acquired with the following parameters: repetition time = 2000 ms, echo time = 20 ms, flip angle = 70°, section thickness = 7 mm, section skip = 0.7 mm, in-plane resolution = 3 × 3 mm. The first 10 volumes were discarded to avoid T1-saturation effects.

### 4.4. Statistical Analyses 

#### 4.4.1. Design

First, family-based genetic association analyses were conducted within EO families and AO families separately. Second, case–control genetic association analyses were tested by comparing HS to EO and AO patients independently. Third, neuroimaging analyses were developed to determine whether brain activity differences exist between HS and subjects with EO and AO SZ depending on the *NRN1* genetic variability.

#### 4.4.2. Genetic Association Analyses

Haploview v4.1 [85] was employed to estimate the linkage disequilibrium (LD) between *NRN1* SNPs. Three haplotype blocks were identified (Block 1: SNP1-SNP3, Block 2: SNP4-SNP5 and Block 3: SNP6-SNP11) in both the family-based and the case–control sample (Supplementary Figure S5). Hardy–Weinberg and genetic association analyses between *NRN1* SNPs/haplotypes and SZ risk/age at onset were conducted using PLINK-v1.07 software [86]. 

For the family-based analyses, SNP and haplotype associations were tested using the Transmission Disequilibrium Test (TDT). This test evaluates whether the transmission frequency of alleles/haplotypes from heterozygous parents to their affected children deviates from the expected Mendelian frequency by comparing the transmitted and not transmitted alleles/haplotypes. 

The case–control analyses were conducted using logistic regressions under different models of inheritance (allelic, genotypic/additive, recessive and dominant), all adjusted by sex. 

In both genetic association approaches, a cut-off threshold for rare haplotypes of 1% and a sliding window approach were applied to the haplotype analyses. The odds ratios (OR) were estimated either from the absolute number of alleles/haplotypes transmitted and not transmitted from parents to affected offspring or from the absolute number of alleles/haplotypes estimated in patients and controls. Multiple testing corrections (10,000 permutations procedure) were applied to all analyses, and all the reported *p*-values are those obtained with this correction (*p_perm_*). As haplotype TDT implemented in PLINK does not include the permutation procedure, to confirm our associations, all the possible haplotypes were reconstructed based on the most likely expectation maximisation (EM) phase and once reconstructed, haplotypic associations were tested using a simple TDT with 10,000 permutations. 

The statistical power was calculated, in the case of the family-based sample, using the ‘trio’ R package version 3.1.2 available at http://www.bioconductor.org, accessed on 1 April 2022. As the sample consisted of 151 trios and the MAF of selected SNPs ranged from 0.20 to 0.50 (Table 1), by assuming an allelic TDT model and a statistical power of 0.80, the smaller detectable relative risk is 1.75. In the case of the case–control sample, the statistical power was calculated using the ‘genpwr’ R package version 1.0.2. As the sample comprised 345 subjects and the MAF of selected SNPs ranged from 0.16 to 0.49, assuming a logistic model and a power of 0.80, the smaller detectable odds ratio is 1.57. 

#### 4.4.3. Neuroimaging Association Study

Based on our genetic association results highlighting the genetic region spanning SNP 6, 7 and 8, we performed the neuroimaging analyses with the haplotype significantly associated with the risk for EO (the HAP678 GTT) in the matched sub-set (39 HS, 39 EO and 39 AO subjects). For these analyses, individuals’ possible haplotype phases were estimated using PLINK and only those with a probability ≥ 95% were included. Because of the haplotypic frequencies in our sample, the analyses were conducted considering the haplotype as a dichotomous variable, and each subject was classified as a non-carrier (0 copies of the risk haplotype) or carrier (1 or 2 copies of the risk haplotype).

Functional MRI pre-processing and analyses were performed with the FEAT tool, from FSL software (FMRIB Software Library, University of Oxford, Oxford, UK; [84]). Pre-processing included motion correction (using the MCFLIRT algorithm with 6 degrees of freedom) and co-registration and normalisation to a common stereotactic space (Montreal Neurological Institute [MNI] template with 2 × 2 × 2 mm resolution) using linear transformations with 12 degrees of freedom. Before group analyses, normalised images were spatially filtered with a Gaussian filter (FWHM = 5mm). To minimise unwanted movement-related effects, individuals with an estimated maximum absolute movement > 3.0 mm or an average absolute movement > 0.3 mm were excluded from analyses.

At the single-subject level analysis, General Linear Models (GLMs) were fitted to generate individual activation maps for each condition of interest compared to baseline and for the comparison between conditions (1-back vs. baseline, 2-back vs. baseline and 2-back vs. 1-back). Temporal derivatives for each condition of interest, as well as movement parameters (six in total, three rotations and three translations), were also included as additional regressors. Fixation periods were not modelled and thus acted as an implicit baseline (i.e., to compare a condition of interest of any given task with its baseline periods, the average BOLD signal from all the baseline periods across the whole task is subtracted from that of the blocks corresponding to the condition of interest). Images were high-pass filtered with a 130 s cut-off. All statistical tests were performed at the cluster level with a corrected *p*-value of 0.05 and an initial height threshold of 3.1 (equivalent to an uncorrected *p*-value of 0.001), using the Standard Field Theory correction implemented in FSL [87].

At the group-level analyses, we studied brain activations and deactivations associated with the execution of the N-back task within each group for all the contrasts, as well as the differences between groups using ANOVA models (two comparisons whole-brain corrected: HS vs. EO/HS vs. AO adjusted for age, sex, and premorbid-IQ).

Since the 1-back requires the maintenance of the target in the memory (keeping track of the target when the consecutive letter is represented) and the 2-back demands both maintenance and target switching (updating the target identity with the appearance of each new letter), we decided to focus our interaction analyses on the 2-back vs. 1-back contrast as it highlights regional responses specific to a higher WM capacity [88,89]. Accordingly, our group differences between HS and patients (both EO and AO), were more pronounced in the 2-back vs. 1-back contrast (Supplementary Figure S5). 

The interaction effect between the diagnosis and the risk haplotype HAP678 GTT was investigated using a regression model, which tests whether the slope between groups and haplotype differs (two models, whole-brain corrected: HS and EO/HS and AO, adjusted for age, sex, and premorbid-IQ). Four contrasts were explored (EO > HS and the reverse contrast; AO > HS and the reverse contrast). Therefore, to control for all the comparisons, the significance threshold used was set to *p* < 0.05/4 = 0.0125. To interpret the direction of the interaction results, we estimated individual mean activity scores from the areas where a significant interaction was detected using the FSLSTATS tool in FSL and afterwards, these values were plotted using SPSS. 

Analyses of the behavioural data (d’1 and d’2) were carried out using SPSS and ANOVA models adjusted for age, sex and estimated IQ. First, N-back task performance was compared between HS and patients (two comparisons: HS vs. EO/HS vs. AO). Second, we explored the effect of the risk haplotype HAP678 GTT on N-back task performance in each group (HS, EO and AO). Third, we tested the interaction between diagnosis and the risk haplotype HAP678 GTT (two models: HS vs. EO/HS vs. AO). Additionally, the change between d’1 and d’2 was explored using repeated measures ANOVA models adjusted for age, sex and estimated IQ. These repeated measures models were conducted, first, between groups (two comparisons: HS vs. EO/HS vs. AO) and, second, between groups depending on the haplotype (two models: HS vs. EO/HS vs. AO).

## 5. Conclusions

Our results contribute to the understanding of the molecular mechanisms underlying SZ early age at onset. Specifically, our work suggests that studying the role of neurotrophins (such as *NRN1*), in specific phenotypes with particularly etiological underpinnings (such as early-onset) and their effect on intermediate phenotypes (such as functional neuroimaging data), helps to elucidate the impact of common genetic variability on biological networks underlying mental disorders.

## Figures and Tables

**Table 1:** Significant genetic association results within early-onset (EO) family-based and EO case–control samples. At the top row, for the family-based approach, there are the transmitted and not-transmitted haplotype (HAP) counts from heterozygous parents to affected offspring for family-based analyses. Below, for the case–control approach, the frequency (%) in healthy subjects (HS) and schizophrenia spectrum disorders (SSD) are given with the risk genotype placed last. The odds ratio (OR) associated with the genotype and the confidence interval (CI 95%) are also reported. The empirical *p*-values obtained after 10,000 permutation procedures (*p_perm_*) for the Transmission Disequilibrium Test (TDT) or the logistic regression (additive model) are shown.

**SNPs**	**Haplotype**	**Transmitted EO SSD**	**Not Transmitted EO SSD**	**OR (CI 95%)**	**TDT; *p_perm_***
HAP678	GCT	13	27	0.48 (0.25–0.93)	4.90; 0.03
**SNPs**	**Genotypes** **Haplotypes**	**Frequency EO SSD**	**Frequency HS**	**OR (CI 95%)**	**Wald; *p_perm_***
SNP6	TT/TG/GG	11 (0.13)/40 (0.48)/33 (0.39)	31 (0.26)/58 (0.49)/30 (0.25)	1.68 (1.01–2.57)	2.39; 0.02 ^a,b^
SNP7	CC/CT/TT	43 (0.50)/35 (0.41)/8 (0.09)	78 (0.66)/35 (0.29)/6 (0.05)	1.69 (1.06–2.71)	2.19; 0.03 ^b^
SNP8	CC/CT/TT	14 (0.17)/41 (0.49)/29 (0.35)	33 (0.29)/58 (0.51)/23 (0.20)	1.66 (1.08–2.56)	2.31; 0.02 ^a,c^
HAP678	TCC	0.37	0.50	0.59 (0.39–0.89)	6.44; 0.01
HAP678	GTT	0.30	0.20	1.70 (1.08–2.67)	5.28; 0.02

^a^ The genotypic model was also significant (*p_perm_* < 0.05). ^b^ The dominant model was also significant (*p_perm_* < 0.05). ^c^ The recessive model was also significant (*p_perm_* < 0.05).

**Table 2:** Sociodemographic and clinical information for the family-based and case-control samples included in the genetic association and neuroimaging analyses. Data for patients are given separately for subjects with early-onset (EO) and adult-onset (AO) schizophrenia spectrum disorders (SSD). Number (percentage in brackets) are shown for qualitative variables. Mean scores (standard deviation in brackets) are provided for quantitative variables. Illness duration refers to years. The psychopathology was assessed using the Positive and Negative Symptoms Scale (PANSS). Treatment was defined by chlorpromazine equivalence (CPZE). Those not significant values are not reported (n.s.).

**Sample 1:** **Family-Based ^a^** **(n = 453)**	**AO Offspring** **(n = 71)**	**EO Offspring** **(n = 80)**		**AO Parents** **(n = 142)**	**EO Parents** **(n = 160)**	
Male	58 (81.70)	54 (67.50)	n.s.	71 (50.00)	80 (50.00)	n.s.
Age at interview	27.45 (5.03)	18.04 (4.94)	t = −11.51, *p* < 0.001	50.04 (7.97)	58.34 (8.44)	t = −7.27, *p* < 0.001
Age at onset	23.24 (4.24)	15.41 (2.12) ^d^	t = −13.35, *p* < 0.001	–	–	–
**Sample 2:** **Case-control ^b^** **(n = 345)**	**AO Subjects** **(n = 138)**	**EO Subjects** **(n = 87)**		**Healthy Subjects** **(n = 120)**		
Male	93 (67.40)	67 (77.00)	n.s.	60 (50.00)		χ^2^ = 17.13, *p* < 0.001
Age at interview	41.97 (10.03)	39.79 (10.87)	n.s.	38.24 (11.21)		F = 3.45; *p* = 0.033 ^e^
Age at onset	25.12 (5.86)	16.38 (2.00)	t = 16.11, *p* < 0.001	–		–
**Sample 3: Neuroimaging ^c^** **(n = 117)**	**AO Subjects** **(n = 39)**	**EO Subjects** **(n = 39)**		**Healthy Subjects** **(n = 39)**		
Male	37 (94.87)	37 (94.87)	n.s.	37 (94.87)		n.s.
Age at interview	39.49 (1.90)	39.30 (1.87)	n.s.	38.43 (1.78)		n.s.
Age at onset	24.56 (0.80)	16.85 (0.26)	t = 9.17; *p* < 0.001	-		-
Illness duration	14.92 (11.01)	22.46 (11.31)	t = −2.98; *p* = 0.004	-		-
PANSS total	68.72 (20.46)	80.05 (21.11)	t = −2.60; *p* = 0.011	-		-
CPZE	367.01 (188.83)	633.66 (304.39)	t = −4.28; *p* < 0.001	-		-

^a^ The different SSD diagnoses were equally distributed between the AO and EO offspring (χ^2^ = 1.271, *p* = 0.736): Schizophrenia (AO n = 41 (57.75%); EO n = 47 (58.75%)), Schizophreniform (AO n = 13 (18.31%); EO n = 10 (12.50%)), Schizoaffective (AO n = 6 (8.45%); EO 7 (8.75%)) and Psychosis not otherwise specified (AO n = 11 (15.4%); EO n = 16 (20.00%)). ^b^ The different diagnoses were equally distributed between the AO and EO cases (χ^2^ = 1.558, *p* = 0.212): Schizophrenia (AO n = 130 (84.42%); EO n = 84 (90.32%)) and Schizoaffective (AO n = 24 (15.58%); EO n = 9 (9.80%)). ^c^ Sub-set of individuals coming from Sample 3. Patients included in the neuroimaging sample were all diagnosed with schizophrenia. ^d^ Information was available for 60% of patients but all were drawn from child and adolescent units which allowed their classification as EO. ^e^ The post-hoc analyses showed significant differences only between healthy subjects and subjects with AO SSD (*p* = 0.010).

**Table 3:** Information on the Single Nucleotide Polymorphisms (SNPs) at Neuritin 1 gene included in this study (*NRN1*, chromosome 6p25.1, from 5,997,999 to 6,007,605 bp, UCSC Genome Browser on Human Assembly GRCh38/hg38, http://genome.ucsc.edu/cgi-bin/hgTracks, accessed on 1 April 2022). The table includes dbSNP number, the chromosome and gene position, the alleles of each SNP, the minor allele frequency (MAF; described for all and EUR populations in the 1000 Genomes Project and the MAF observed in each sample included in the present study) and the functional score according to the Regulome Database.

SNPs		Chromosome Position	Gene Position	Alleles (Minor/Major)	MAF (All/Eur)	Family-Based MAF	Case-Control MAF	RegulomeDB Score ^a^
SNP1	rs2208870	5,992,257	intergenic	G/A	0.33/0.34	0.33	0.33	0.61
SNP2	rs12333117	5,994,759	intergenic	T/C	0.35/0.40	0.43	0.38	0.61
SNP3	rs582186	6,001,148	downstream	A/G	0.45/0.62	0.61	0.40	0.61
SNP4	rs645649	6,004,726	intronic	C/G	0.45/0.64	0.64	0.38	0.61
SNP5	rs582262	6,007,758	intronic	C/G	0.30/0.48	0.28	0.27	0.70
SNP6	rs3763180	6,009,615	upstream	T/G	0.40/0.46	0.45	0.43	0.63
SNP7	rs10484320	6,010,204	upstream	T/C	0.15/0.22	0.26	0.24	0.16
SNP8	rs4960155	6,010,306	upstream	T/C	0.43/0.49	0.50	0.49	0.13
SNP9	rs9379002	6,012,158	intergenic	G/T	0.29/0.42	0.24	0.26	0.13
SNP10	rs9405890	6,012,488	intergenic	C/T	0.31/0.38	0.28	0.33	0.18
SNP11	rs1475157	6,016,936	intergenic	G/A	0.16/0.17	0.16	0.16	0.18

^a^ This score ranges from 0 to 1, with 1 being most likely to be a regulatory variant.

## Supplementary Materials

The following supporting information can be downloaded at: https://www.mdpi.com/article/10.3390/ijms23137456/s1.
