# Supplementary material for: NRN1 Gene as a Potential Marker of Early-Onset Schizophrenia: Evidence from Genetic and Neuroimaging Approaches
Source: Int J Mol Sci. 2022 Jul 5;23(13):7456. doi: 10.3390/ijms23137456 (PMC9267632; doi:10.3390/ijms23137456)
Supplement: Supplementary file 1 [file ijms-23-07456-s001.zip › ijms-1728231-supplementary.pdf]

SUPPLEMENTARY TABLES

Supplementary Table S1. Allelic and genotypic data of offspring with schizophrenia-spectrum disorder (SSD) and their healthy parents included in the family-based sample. For each SNP the table includes the dbSNP, the allele and the genotypes count and frequency (%), the statistic corresponding to allelic and genotypic group comparison. As observed, the statistical comparison (X2; p-value) indicate that allelic and genotypic distributions did not differ between EO and AO offspring nor EO and AO parents.

NRN1 SNPs	Alleles and Genotypes[a]	Subjects with SSD		χ^2; p-value	Healthy Subjects		χ^2; p-value
		Adult-Onset Offspring (n=71)	Early-Onset Offspring (n=80)		Adult-Onset Parents (n=142)	Early-Onset Parents (n=160)	
rs2208870	G/A	46(32.4) / 96(67.6)	51(34) / 99(66)	0.08; 0.77	89(31.6) / 193(68.4)	98(33.8) / 192(66.2)	0.32; 0.57
	GG/GA/AA	3(4.2) / 40(56.3) / 28(39.4)	8(10.7) / 35(46.7) / 32(42.7)	2.76; 0.25	10(7.1) / 69(48.9) / 62(44)	20(13.8) / 58(40.0) / 67(46.2)	4.42; 0.11
rs12333117	T/C	57(40.7) / 83(59.3)	69(47.3) / 77(52.7)	1.24; 0.26	113(40.1) / 169(59.9)	126(44.1) / 160(55.9)	0.92; 0.34
	TT/TC/CC	11(15.7) / 35(50) / 24(34.3)	16(21.9) / 37(50.7) / 20(27.4)	1.28; 0.53	23(16.3) / 67(47.5) / 51(36.2)	31(21.7) / 64(44.8) / 48(33.6)	1.33; 0.51
rs582186	G/A	56(40.0) / 84(60.0)	51(38.1) / 83(61.9)	0.11; 0.74	111(40.5) / 163(59.5)	106(37.1) / 180(62.9)	0.70; 0.40
	GG/GA/AA	7(10.0) / 42(60.0) / 21(30.0)	11(16.4) / 29(43.3) / 27(40.3)	3.96; 0.14	26(19.0) / 59(43.1) / 52(38.0)	24(16.8) / 58(40.6) / 61(42.7)	0.68; 0.71
rs645649	G/C	53(37.9) / 87(62.1)	53(36.3) / 93(63.7)	0.07; 0.78	94(34.6) / 178(65.4)	101(35.6) / 183(64.4)	0.06; 0.80
	GG/GC/CC	8(11.4) / 37(52.9) / 25(35.7)	9(12.3) / 35(47.9) / 29(39.7)	0.35; 0.84	17(12.5) / 60(44.1) / 59(43.4)	20(14.1) / 61(43.0) / 61(43.0)	0.15; 0.92
rs582262	C/G	45(32.1) / 95(67.9)	39(27.5) / 103(72.5)	0.74; 0.39	78(28.3) / 198(71.7)	76(27.0) / 206(73.0)	0.12; 0.73
	CC/CG/GG	8(11.4) / 29(41.4) / 33(47.1)	6(8.5) / 27(38) / 38(53.5)	0.70; 0.70	15(10.9) / 48(34.8) / 75(54.3)	11(7.8) / 54(38.3) / 76(53.9)	0.94; 0.62
rs3763180	T/G	58(42.6) / 78(57.4)	71(48.6) / 75(51.4)	1.02; 0.31	114(42.5) / 154(57.5)	141(47.0) / 159(53.0)	1.34; 0.28
	TT/TG/GG	13(19.1) / 32(47.1) / 23(33.8)	14(19.2) / 43(58.9) / 16(21.9)	2.73; 0.25	28(20.9) / 58(43.3) / 48(35.8)	31(20.7) / 79(52.7) / 40(26.7)	3.21; 0.20
rs10484320	T/C	34(24.3) / 106(75.7)	40(26.7) / 110(73.3)	0.22; 0.64	71(25.7) / 205(74.3)	84(27.8) / 218(72.2)	0.32; 0.57
	TT/TC/CC	2(2.9) / 30(42.9) / 38(54.3)	4(5.3) / 32(42.7) / 39(52)	0.57; 0.75	12(8.7) / 47(34.1) / 79(57.2)	7(4.6) / 70(46.4) / 74(49)	5.43; 0.07
rs4960155	C/T	69(49.3) / 71(50.7)	64(43.8) / 82(56.2)	0.85; 0.36	130(46.8) / 148(53.2)	147(49.0) / 153(51.0)	0.29; 0.59
	CC/CT/TT	17(24.3) / 35(50) / 18(25.7)	13(17.8) / 38(52.1) / 22(30.1)	0.99; 0.61	33(23.7) / 64(46.0) / 42(30.2)	36(24) / 75(50) / 39(26)	0.69; 0.71
rs9379002	G/T	37(26.4) / 103(73.6)	26(17.8) / 120(82.2)	3.09; 0.08	73(26.3) / 205(73.7)	66(22.1) / 232(77.9)	1.33; 0.25
	GG/GT/TT	5(7.1) / 27(38.6) / 38(54.3)	2(2.7) / 22(30.1) / 49(67.1)	3.12; 0.21	13(9.4) / 47(33.8) / 79(56.8)	10(6.7) / 46(30.9) / 93(62.4)	1.19; 0.55
rs9405890	C/T	40(28.2) / 102(71.8)	39(24.4) / 121(75.6)	0.56; 0.45	81(28.5) / 203(71.5)	88(27.7) / 230(72.3)	0.05; 0.82
	CC/CT/TT	3(4.2) / 34(47.9) / 34(47.9)	3(3.8) / 33(41.3) / 44(55)	0.76; 0.68	8(5.6) / 65(45.8) / 69(48.6)	10(6.3) / 68(42.8) / 81(50.9)	0.29; 0.86
rs1475157	G/A	24(16.9) / 118(83.1)	23(15.1) / 129(84.9)	0.17; 0.68	49(17.3) / 235(82.7)	47(15.0) / 267(85.0)	0.58; 0.45
	GG/GA/AA	2(2.8) / 20(28.2) / 49(69)	1(1.3) / 21(27.6) / 54(71.1)	0.43; 0.81	5(3.5) / 39(27.5) / 98(69.0)	6(3.8) / 35(22.3) / 116(73.9)	1.07; 0.58

[a] Minor allele is placed first. The homozygote for the minor allele is placed first followed by the heterozygote and the homozygote for the major allele.

Supplementary Table S2
Allelic and genotypic data of the cases with schizophrenia-spectrum disorder (SSD) and the healthy controls included in the case-control sample. For each SNP the table includes the dbSNP, the allele and the genotypes count and frequency (%) and the statistic corresponding to allelic and genotypic group comparison.

NRN1 SNPs	Alleles and Genotypes [a]	SSD Subjects		Healthy Subjects
		Adult-Onset Cases (n=138)	Early-Onset Cases (n=87)	Healthy Controls (n=120)
rs2208870	G/A	96(34.8) / 180(65.2)	56(32.2) / 118(67.8)	80(33.3) / 160(66.7)
	GG/GA/AA	16(13.3) / 64(53.3) / 58(48.3)	11(9.2) / 34(28.3) / 42(35)	10(8.3) / 60(50) / 50(41.7)
rs12333117	T/C	97(35.4) / 177(64.6)	70(40.7) / 102(59.3)	88(37.3) / 148(62.7)
	TT/TC/CC	17(14.2) / 63(52.5) / 57(47.5)	16(13.3) / 38(31.7) / 32(26.7)	20(16.7) / 48(40) / 50(41.7)
rs582186	A/G	120(44.1) / 152(55.9)	66(38.8) / 104(61.2)	92(38.7) / 146(61.3)
	AA/AG/GG	24(20) / 72(60) / 40(33.3)	15(12.5) / 36(30) / 34(28.3)	16(13.3) / 60(50) / 43(35.8)
rs645649	C/G	110(39.9) / 166(60.1)	60(35.3) / 110(64.7)	92(38.7) / 146(61.3)
	CC/CG/GG	22(18.3) / 66(55) / 50(41.7)	9(7.5) / 42(35) / 34(28.3)	15(12.5) / 62(51.7) / 42(35)
rs582262	C/G	81(29.8) / 191(70.2)	37(22.6) / 127(77.4)	69(29.2) / 167(70.8)
	CC/CG/GG	14(11.7) / 53(44.2) / 69(57.5)	3(2.5) / 31(25.8) / 48(40)	11(9.2) / 47(39.2) / 60(50)
rs3763180	T/G	108(39.4) / 166(60.6)	62(36.9) / 106(63.1)	120(50.4) / 118(49.6)
	TT/TG/GG	22(18.3) / 64(53.3) / 51(42.5)	11(9.2) / 40(33.3) / 33(27.5)	31(25.8) / 58(48.3) / 30(25)
rs10484320	T/C	75(27.2) / 201(72.8)	51(29.7) / 121(70.3)	47(19.7) / 191(80.3)
	TT/TC/CC	7(5.8) / 61(50.8) / 70(58.3)	8(6.7) / 35(29.2) / 43(35.8)	6(5) / 35(29.2) / 78(65)
rs4960155	C/T	145(52.5) / 131(47.5)	69(41.1) / 99(58.9)	124(54.4) / 104(45.6)
	CC/CT/TT	34(28.3) / 77(64.2) / 27(22.5)	14(11.7) / 41(34.2) / 29(24.2)	33(27.5) / 58(48.3) / 23(19.2)
rs9379002	G/T	69(25.2) / 205(74.8)	49(28.8) / 121(71.2)	61(25.6) / 177(74.4)
	GG/GT/TT	8(6.7) / 53(44.2) / 76(63.3)	7(5.8) / 35(29.2) / 43(35.8)	8(6.7) / 45(37.5) / 66(55)
rs9405890	C/T	107(38.8) / 169(61.2)	54(31) / 120(69)	70(29.2) / 170(70.8)
	CC/CT/TT	16(13.3) / 75(62.5) / 47(39.2)	10(8.3) / 34(28.3) / 43(35.8)	8(6.7) / 54(45) / 58(48.3)
rs1475157	G/A	38(13.8) / 238(86.2)	36(20.7) / 138(79.3)	37(15.4) / 203(84.6)
	GG/GA/AA	3(2.5) / 32(26.7) / 103(85.8)	4(3.3) / 28(23.3) / 55(45.8)	6(5) / 25(20.8) / 89(74.2)

[a] For the allelic distribution the minor allele is placed first. For the genotypic distribution the homozygote for the minor allele is placed first followed by the heterozygote and the homozygote for the major allele.

Supplementary Table S3

Other significant genetic association results for the EO vs HS comparison. The frequency (%) in subjects with schizophrenia spectrum disorders (SSD) and healthy subjects (HS), the odds ratio (OR) and the confidence interval (CI 95%) and the empirical p-values obtained after 10000 permutation procedure (p_{perm}) for the logistic regression are given.

SNP / Haplotype	Genotype	Frequency EO SSD	Frequency HS	OR (CI 95%)	Wald; p_{perm}
HAP67	GT	0.30	0.19	1.73 (1.09 – 2.73)	5.51; 0.02
HAP67	TC	0.36	0.50	0.54 (0.35 – 0.82)	8.15; 0.002
HAP78	CC	0.40	0.55	0.55 (0.36 – 0.83)	7.91; 0.004
HAP78	TT	0.30	0.19	1.72 (1.09 – 2.72)	5.36; 0.02
HAP678	GTT	0.30	0.20	1.70 (1.08 – 2.67)	5.28; 0.02

SUPPLEMENTARY FIGURES

A) Activations and deactivations within healthy subjects.

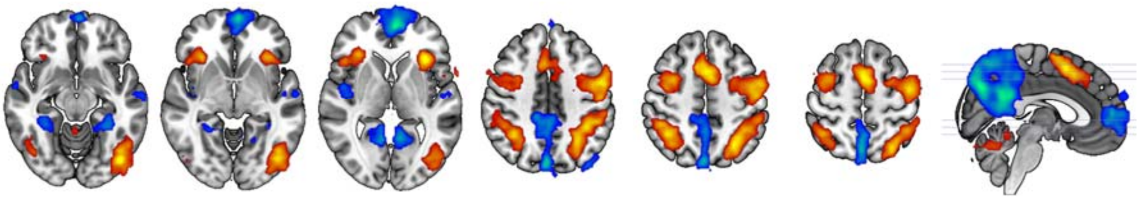

B) Activations and deactivations within subjects with adult-onset schizophrenia.

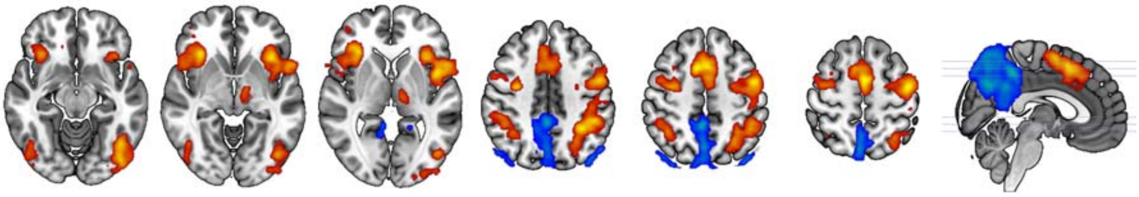

C) Activations and deactivations within subjects with early-onset schizophrenia.

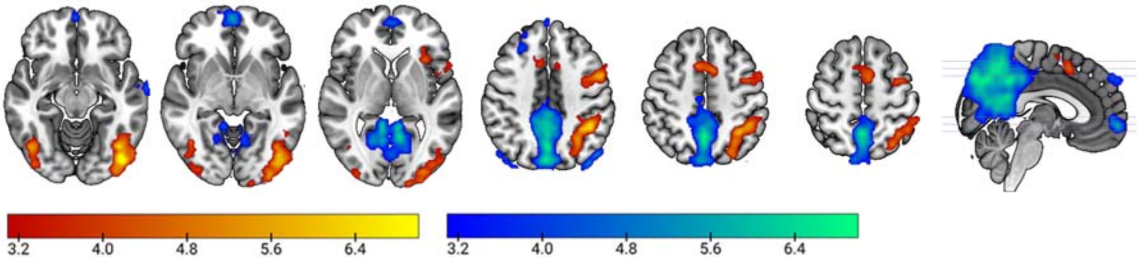

Supplementary Figure S1. Brain activation and deactivation maps for the 1-back vs baseline contrast. A typical working-memory related brain activity for healthy subjects **(A)**, adult-onset **(B)** and early-onset subjects **(C)**. All images correspond to the axial view of the brain. The sagittal view of the brain is also included with the mark of the cross slices. The right side of the image represents the right side of the brain. MNI coordinates are given for the shown slices. Units of the bar are the corresponding β values from the regression standardised to Z scores. Calid colours represent activations associated with task performance while cold colours represent deactivations. All clusters are significant (p<0.05).

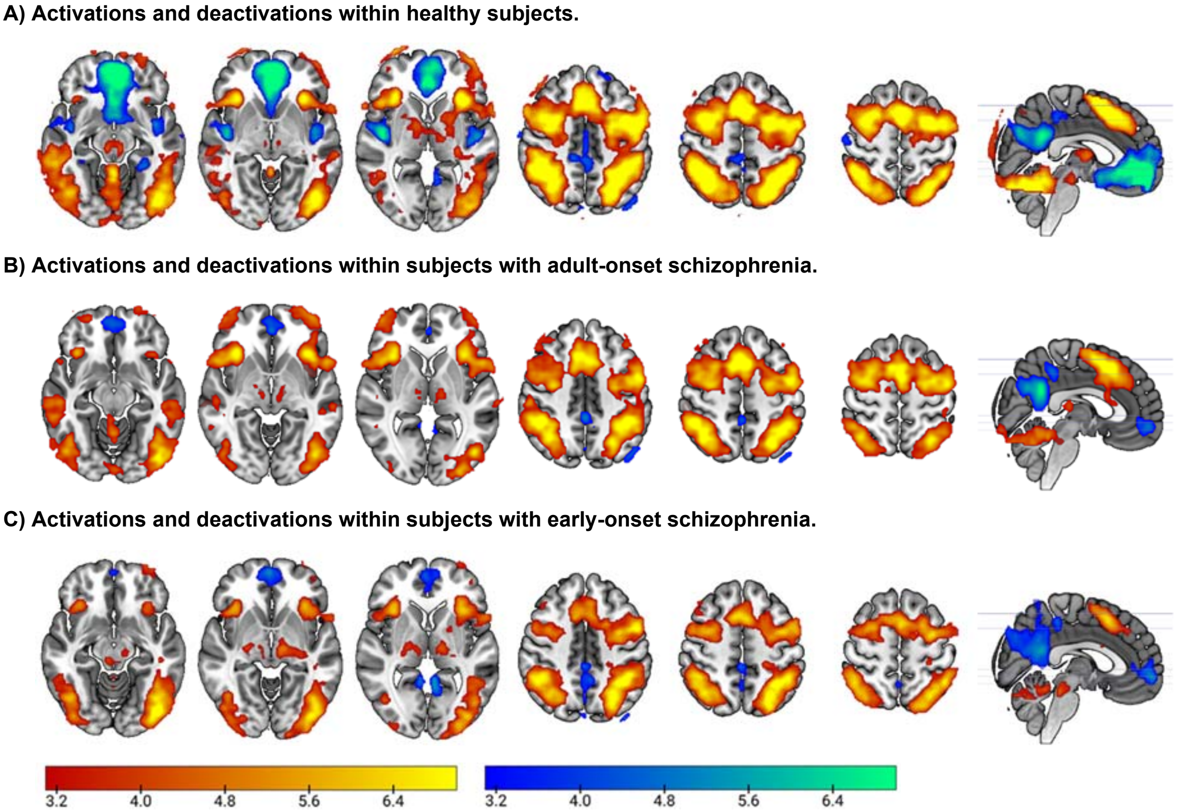

Supplementary Figure S2. Brain activation and deactivation maps for the 2-back vs baseline contrast. A typical working-memory related brain activity for healthy subjects **(A)**, adult-onset **(B)** and early-onset subjects **(C)**. All images correspond to the axial view of the brain. The sagittal view of the brain is also included with the mark of the cross slices. The right side of the image represents the right side of the brain. MNI coordinates are given for the shown slices. Units of the bar are the corresponding β values from the regression standardised to Z scores. Calid colours represent activations associated with task performance while cold colours represent deactivations. All clusters are significant ($p<0.05$).

A) Activations and deactivations within healthy subjects.

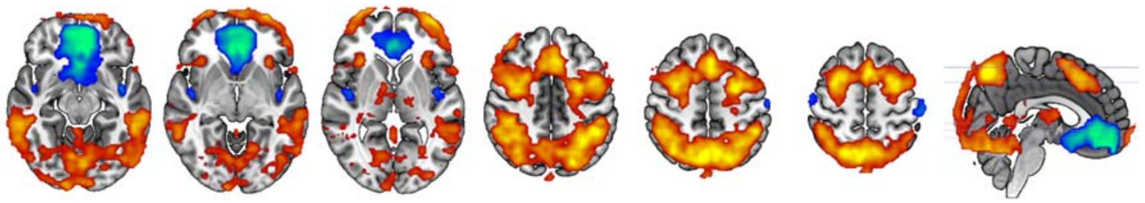

B) Activations and deactivations within subjects with adult-onset schizophrenia.

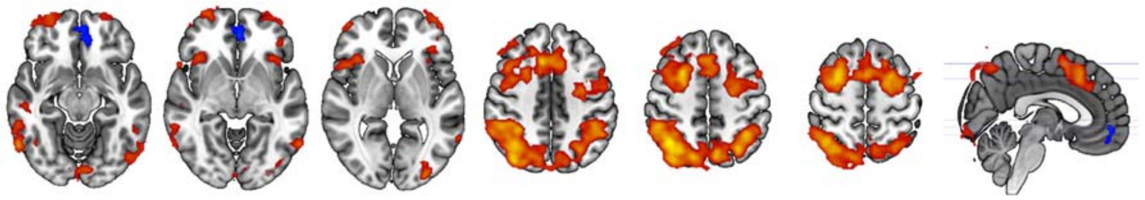

C) Activations and deactivations within subjects with early-onset schizophrenia.

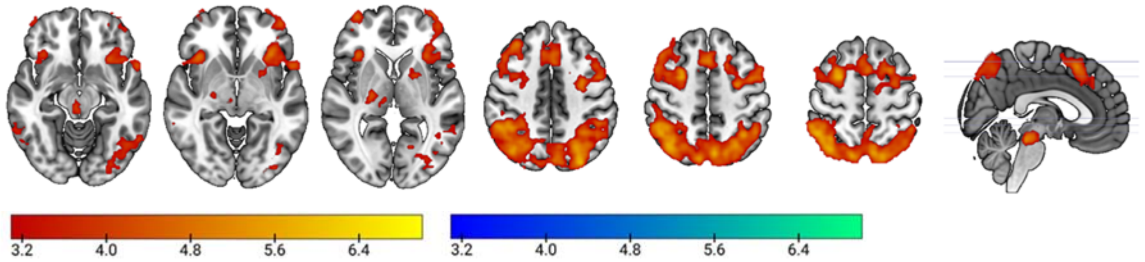

Supplementary Figure S3. Brain activation and deactivation maps for the 2-back vs 1-back contrast. A typical working-memory related brain activity for healthy subjects **(A)**, adult-onset **(B)** and early-onset subjects **(C)**. All images correspond to the axial view of the brain. The sagittal view of the brain is also included with the mark of the cross slices. The right side of the image represents the right side of the brain. MNI coordinates are given for the shown slices. Units of the bar are the corresponding β values from the regression standardised to Z scores. Calid colours represent activations associated with task performance while cold colours represent deactivations. All clusters are significant (p<0.05).

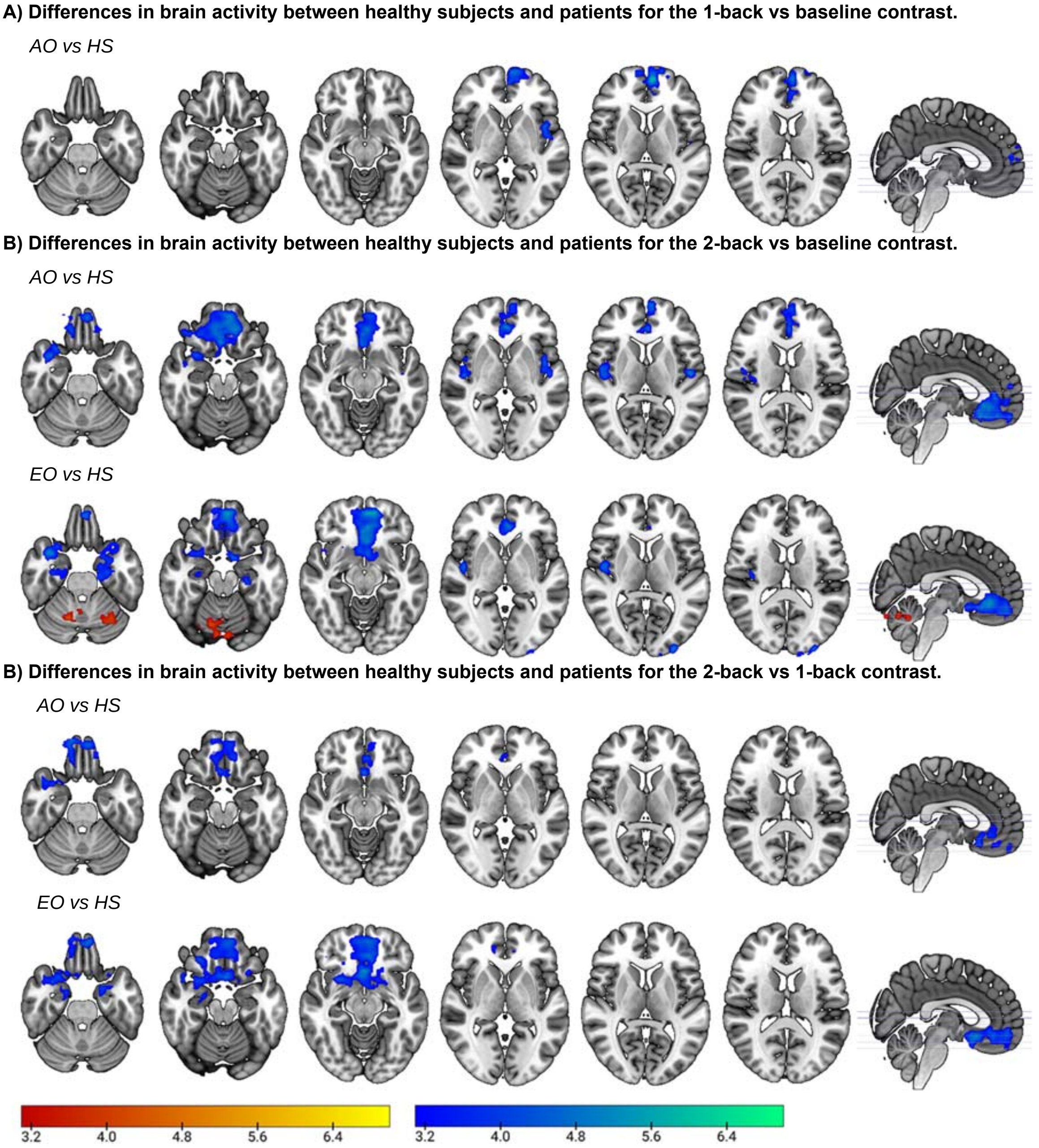

Supplementary Figure S4. Brain regions showing significant differences between patients and controls. The image at the top represents areas differentially activated in the 1-back vs baseline contrast **(A)**, the image at the middle in the 2-back vs baseline contrast **(B)** and the image at the bottom in the 2-back vs 1-back contrast **(C)**. All images correspond to the axial view of the brain. The sagittal view of the brain is also included with the mark of the cross slices. The right side of the image represents the right side of the brain. MNI coordinates are given for the shown slices. Units of the bar are the corresponding β values from the regression standardised to Z scores. Calid colours represent areas where controls activate more than patients while cold colours represent areas where patients activate more than controls. All clusters are significant ($p<0.05$). HS: healthy subjects; AO: adult-onset; EO: early-onset.

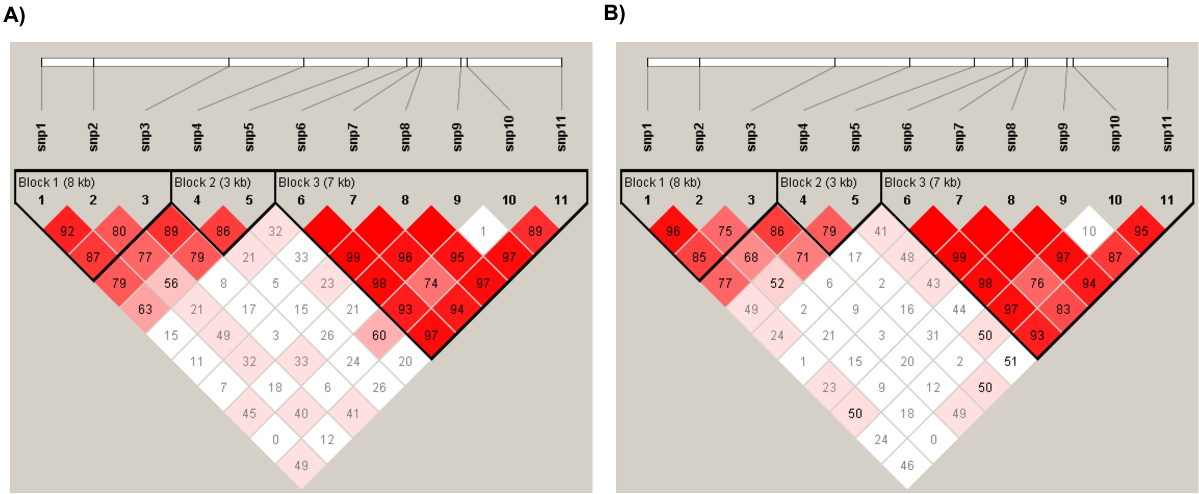

Supplementary Figure S5. Pairwise linkage disequilibrium (LD) estimates (expressed in D′) between the *NRN1* markers genotyped in the family-based **(A)** and the case-control samples **(B)** showing the three haplotypic blocks (Haploview 4.1). LD is the condition in which the haplotype frequencies in a population deviate from the values they would have if the SNPs at each locus were combined at random. The higher the D' value, the more non-random is the association between the alleles at different loci. Red-coded squares contain D' values, which are on a scale from 1 to 100; if there is no value, D' = maximum.
